# A review of visualization techniques of post-mortem computed tomography data for forensic death investigations

**DOI:** 10.1007/s00414-021-02581-4

**Published:** 2021-04-30

**Authors:** Lars Christian Ebert, Sabine Franckenberg, Till Sieberth, Wolf Schweitzer, Michael Thali, Jonathan Ford, Summer Decker

**Affiliations:** 1 Zurich Institute of Forensic Medicine, University of Zurich, Zurich, Switzerland; 2 Institute of Diagnostic and Interventional Radiology, University Hospital Zurich, Zurich, Switzerland; 3 Department of Radiology, University of South Florida, Morsani College of Medicine, Tampa, FL USA

**Keywords:** Postmortem computed tomography, Visualization, Virtual reality, Segmentation, 3D printing, Reporting

## Abstract

Postmortem computed tomography (PMCT) is a standard image modality used in forensic death investigations. Case- and audience-specific visualizations are vital for identifying relevant findings and communicating them appropriately. Different data types and visualization methods exist in 2D and 3D, and all of these types have specific applications. 2D visualizations are more suited for the radiological assessment of PMCT data because they allow the depiction of subtle details. 3D visualizations are better suited for creating visualizations for medical laypersons, such as state attorneys, because they maintain the anatomical context. Visualizations can be refined by using additional techniques, such as annotation or layering. Specialized methods such as 3D printing and virtual and augmented reality often require data conversion. The resulting data can also be used to combine PMCT data with other 3D data such as crime scene laser scans to create crime scene reconstructions. Knowledge of these techniques is essential for the successful handling of PMCT data in a forensic setting. In this review, we present an overview of current visualization techniques for PMCT.

## Introduction

Forensic cases are increasingly being documented using 3D imaging of both humans (victims, perpetrators) and objects (crime scenes, vehicles, weapons) using a wide variety of scanning techniques [1]. For postmortem imaging, x-ray, computed tomography (CT), CT angiography, magnetic resonance imaging (MRI), and various surface scanning methods are utilized to investigate the circumstances of death of a deceased and to document the findings [2–5]. In such instances, postmortem CT (PMCT) is the preferred modality; it permits assessment of most forensically relevant conditions such as bone fractures, hemorrhage, parenchymal lacerations, free intracorporal gas accumulation, and the presence of foreign bodies [6, 7]. PMCT scanning protocols can be optimized for use in forensic cases to increase image quality by using higher energies, thus lowering image noise [2]. In addition, clinical CT scans can be reevaluated forensically for potential use in criminal proceedings. CT scans can also help in 3D documenting objects [8, 9].

PMCT generates volumetric data that can be used in a variety of applications in forensic sciences. First, the data can be used as a triage tool for autopsy [10], to thoroughly plan a subsequent autopsy [11] and to assist in determining cause and manner of death [6]. Second, PMCT can be utilized to reconstruct the course of events [12, 13], to create comprehensible visualizations for presentation to state attorneys and judges [14], and to add information on the internal body state of the deceased, such as the presence of underlying medical conditions or gunshot trajectories, to 3D-surface scanning [15–17]. This mix of different image modalities adds resolution and color information to the CT scan, which would otherwise be insufficient for the assessment of would surface morphology. Third, it can be used to identify bodies, i.e., in cases of mass disasters [18, 19], thus potentially speeding up disaster victim identification scenarios. Other fields of application include anthropological analyses such as sex or age estimation and identification [20] and medical training [21].

Various forensic disciplines, including forensic medicine, forensic anthropology, and forensic crime scene reconstruction, routinely work with postmortem image data [7, 16, 22]. Forensic radiologists analyze the images for initial diagnosis, and forensic pathologists are able to plan autopsies based on those findings [2]. Both clinical and forensic radiologists primarily use 2D slices rather than 3D visualization in the assessment of CT data to perceive subtle details that might otherwise be missed [23]. However, for adequate interpretation of these 2D images of PMCT datasets, precise knowledge of anatomy, awareness of regular postmortem alterations, spatial imagination, and knowledge of the basic operating principles of the examination modality are required. Lacking this type of specialized knowledge, medical laypersons such as prosecutors, lawyers, and judges rely on more comprehensible visualizations of the findings. This information is often provided to them in the form of a printed image report containing annotated 2D images as well as 3D renderings or dedicated 3D prints as a supplement [2, 24]. In addition, police experts such as forensic officers and technicians can imbed the data and findings from PMCT into their crime scene reconstructions through additional processing steps [15].

Visualization of forensic medical data can be confusing at first due to the large variety of techniques, image modalities, and software available. For every visualization task described in this article, free or open-source software that can be used at no additional cost is available (Table 1). While commercial software products often feature streamlined graphical user interfaces and cutting-edge visualization features to increase their commercial success, they are often quite expensive [25]. In contrast, for those with limited budget, free open-source software may be more affordable and allow independence of vendors, and in some instances, it provides excellent performance [26, 27]. However, free software can come with comparative weaknesses such as insufficient stability, an inferior graphical user interface, and thus a requirement for advanced user skills, lack of support, lack of certification, and lack of accountability, thus ultimately leading to higher costs [28].

**Table 1:** List of free and open-source software that can be used in different visualization tasks

Task	Software	Website
2D visualization	Horos	https://horosproject.org/
2D annotation 2D Bitmap editing/layering	Gimp	https://www.gimp.org/
2D annotation	Inkscape	https://inkscape.org/
	LibreOffice Impress	https://www.libreoffice.org/
3D VRT	Horos	https://horosproject.org/
	3D Slicer	https://www.slicer.org/
3D VRT, Physical Shader	MeVisLab	https://www.mevislab.de/
Segmentation	3D Slicer	https://www.slicer.org/
3D polygon processing	MeshLab	http://www.meshlab.net/
3D polygon rendering	Blender	https://www.blender.org/
Virtual reality	Unity	https://unity.com/
2D Bitmap editing	Gimp	https://www.gimp.org/
3D printing	Slic3r	https://slic3r.org/

In this review article, we introduce different methods of visualizing PMCT in 2D and 3D and give examples on how to use these techniques, based on the current scientific literature. We show how to convert PMCT data to polygon models that are suitable for 3D printing, metrology, and 3D reconstruction. We limit this review to techniques that are available in widely used software. We also provide a list of open-source and free software tools to give a starting point for the visualization of PMCT data. The review is structured in two main components: 2D and 3D visualizations. First, for the 2D section, data types used in conjunction with visualizing PMCT data are introduced. This is followed by 2D visualization techniques, as they are often used for analyzing PMCT images radiologically. For the 3D section, 3D visualization methods are discussed, following a workflow that is often used when visualizing in 3D - first, direct volume visualization using volume rendering, followed by the generation of polygon models through segmentation for specialized visualization applications. Finally, different visualization options for polygonal data such as 3D printing or virtual reality/augmented reality are introduced.

## Visualization techniques

### Data types

The Digital Imaging and Communication in Medicine (DICOM) standard is the standard format for storing digital medical images [29]. An image stored in DICOM format consists of two parts—the image data and a “header” containing information such as patient data and the scanning protocol used. 3D volumetric data such as CT scans are stored in stacks of images contained in separate DICOM files. A CT volume consists of single elements called volume picture elements (voxels), which are similar to picture elements (pixels) in a 2D image. Each voxel has a position in the volume and a value depending on the image modality; in the case of CT data, the value is the x-ray attenuation measured in Hounsfield units (HU; for more detailed information, see the following section) [30].

**Fig. 1:**
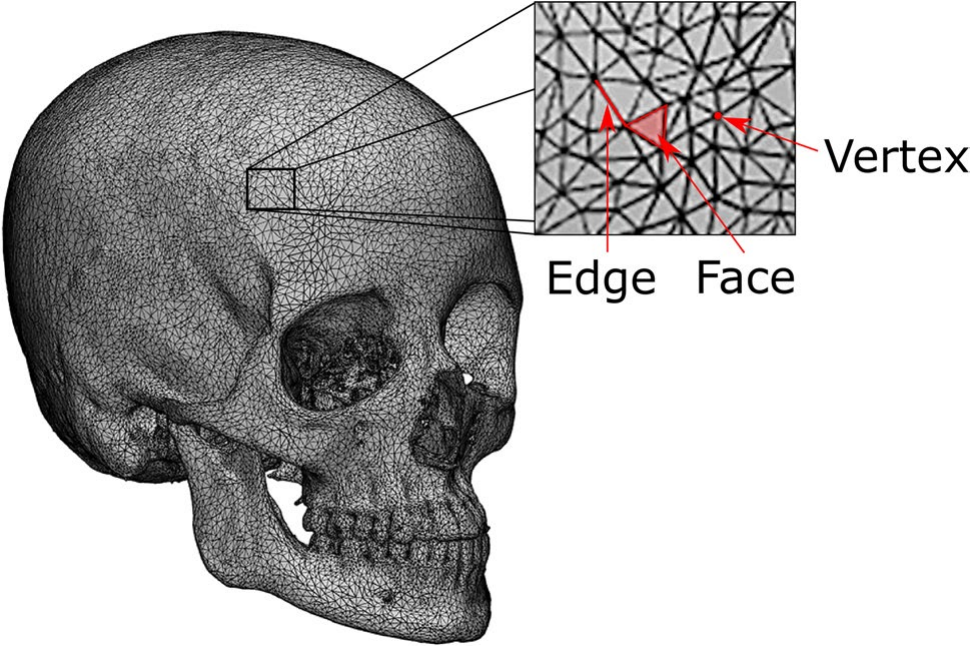
Polygon model describing the surface of an anthropologist’s skull, based on data extracted from a CT scan. Polygon meshes consist of points called vertexes that are interconnected by their edges to form polygons or faces

While voxels are the standard for visualizing medical images, many software packages, such as the ones used in crime scene reconstruction or 3D printing, require polygon meshes. Polygon meshes consist of 3D points called vertexes that are interconnected through edges to form polygons (Fig. 1). While volumetric data consisting of voxels describe the entire volume of an object, including its insides, polygon meshes are used to describe the shape of an object [31]. Using segmentation, volume data can be converted into polygon meshes.

### 2D visualization

**Fig. 2:**
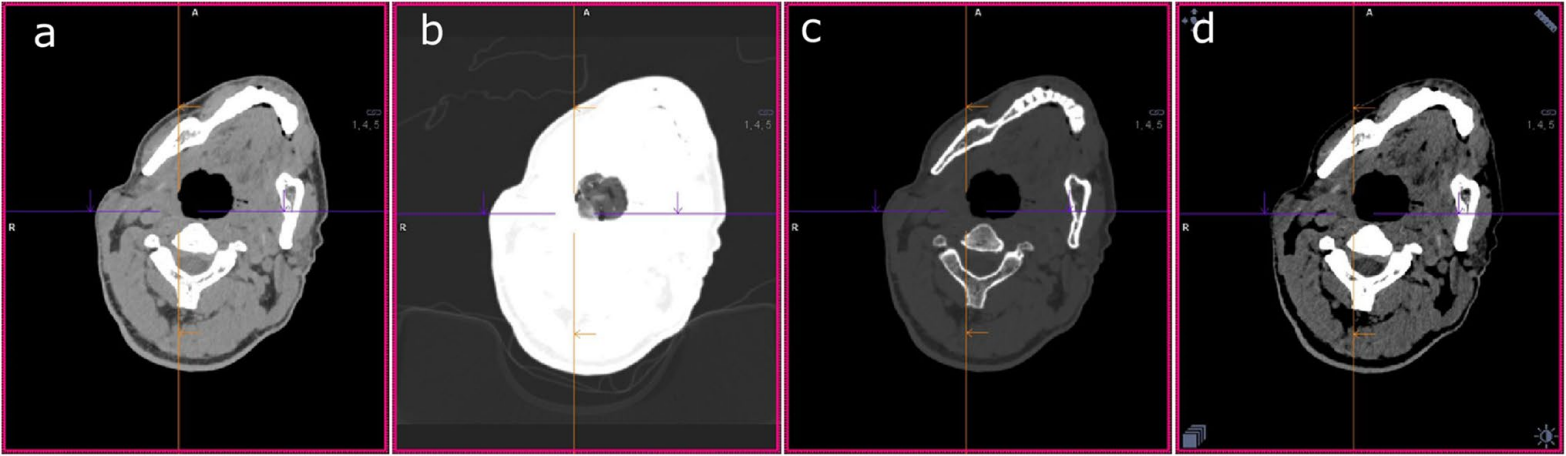
PMCT scan of the neck, visualized in different windows. (**a**) soft tissue window; (**b**) lung window; (**c**) bone window; (**d**) brain window. Note that the material obstructing the airways is only visible in image b, the lung window, and does not appear in any of the other images

HU are a measure of x-ray attenuation at a specific location, with increasing values signifying more attenuation [32]. The Hounsfield scale ranges from -1000 for gases to 0 for water to several thousand HU for metals. HU are usually stored using 12 bits per voxel, which means that every voxel can have one of 4096 different values. Because standard computer screens are only able to display a limited number of shades of gray (typically 256), CT datasets cannot be displayed directly, and further processing is required. Typically, this is done by windowing [33]. A range (the window) of HU that most likely contains the structures of interest is selected and mapped to the 256 available grayscale values. All voxels with values below the lower limit of the selected window are displayed as black, and all voxels with values above its upper limit are displayed as white. A window consists of two values, a defined position on the HU histogram and a width. Some structures and pathologies are only perceivable in a specific window. An example of displays obtained using different window settings and the same dataset can be seen in Fig. 2.

### Multiplanar and curved reformations

**Fig. 3:**
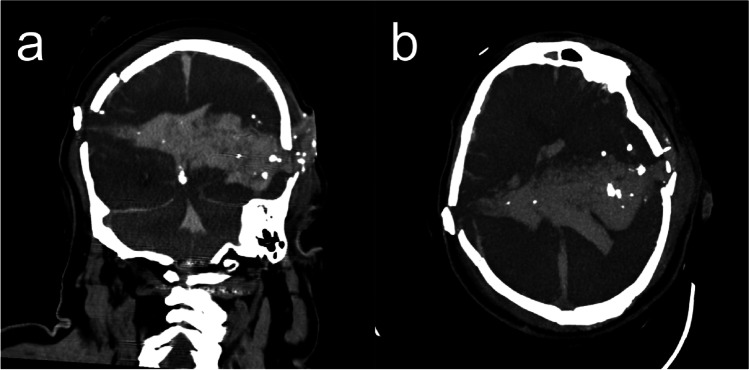
MPR of a suicidal gunshot injury with the entry wound on the right and the exit wound on the left side. The reconstruction follows the path of the bullet through the brain. (**a**) coronal view; (**b**) axial view

**Fig. 4:**
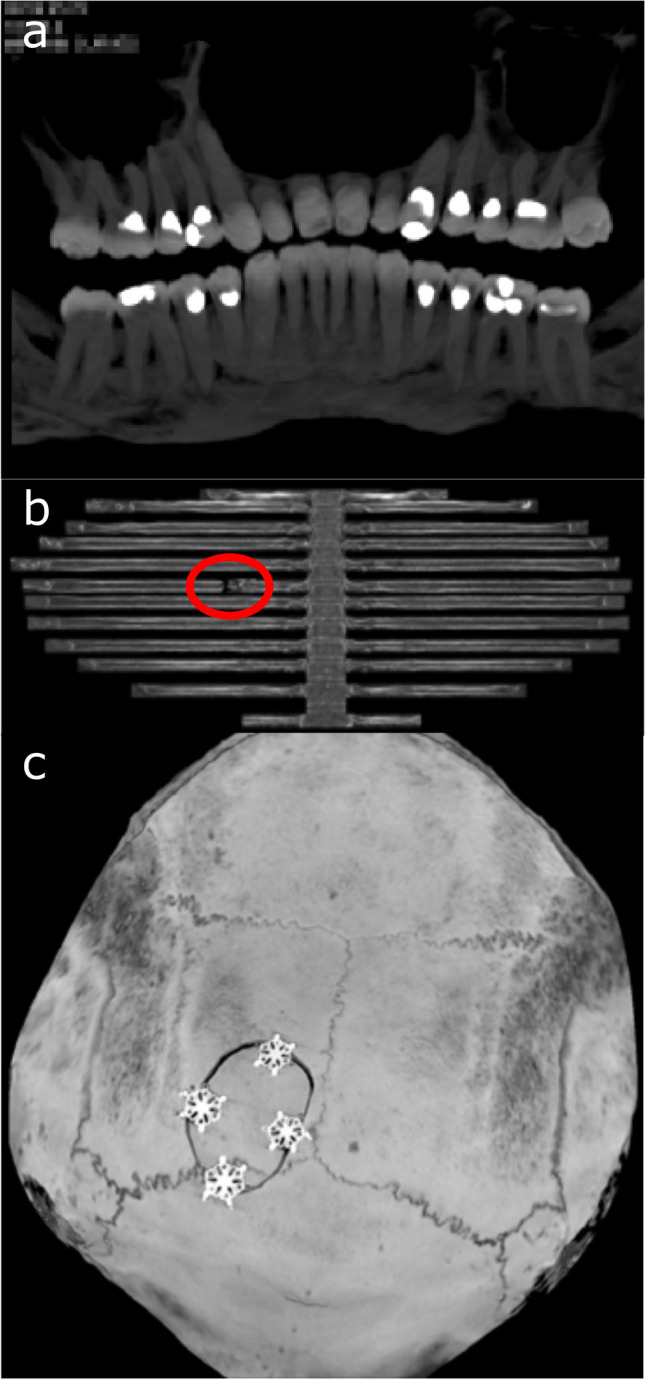
Different types of complex 2D reconstructions. **a** Curved MPR of an individual’s dentition, simulating an orthopantomogram that can be compared to antemortem images. **b** Rib unfolding that can be used in the rapid assessment of multiple rib fractures (**c**). Skull unfolding showing surgical plates and screws after surgical intervention

To improve the assessment of certain anatomical regions and pathologies, acquired data, mostly from the axial plane, can be reformatted to nonaxial planes in a so-called multiplanar reformation (MPR) (Fig. 3). For an MPR, the 3D volume is resliced into planes that can be arbitrarily tilted and positioned. To avoid stairstep artifacts, the scan resolution must be sufficiently high in all directions [34]. MPR is one of the basic techniques that is used to assess CT images and to visualize findings in forensic radiology; among other applications, it can be used in the reconstruction of gunshot wounds [35], the visualization of foreign bodies [36], and victim identification [37]. As a subcategory of MPR, curved MPR is also available. In curved MPR, instead of reformatting the CT data along a chosen plane, a 2D image is generated along a selected curve (e.g., such as a coronary artery), which results in clearer depictions of certain anatomical structures and pathologies that would be difficult to comprehend when viewed in a plane. In the forensic context, MPR is, for example, used in the visualization of the entire skull in one image [38] (Fig. 4), in the visualization of the whole rib cage to assess rib fractures [39] and in the creation of a virtual orthopantomogram by following the contours of the dental arches [40].

### Maximum and minimum intensity projections (MIP, MinIP)

**Fig. 5:**
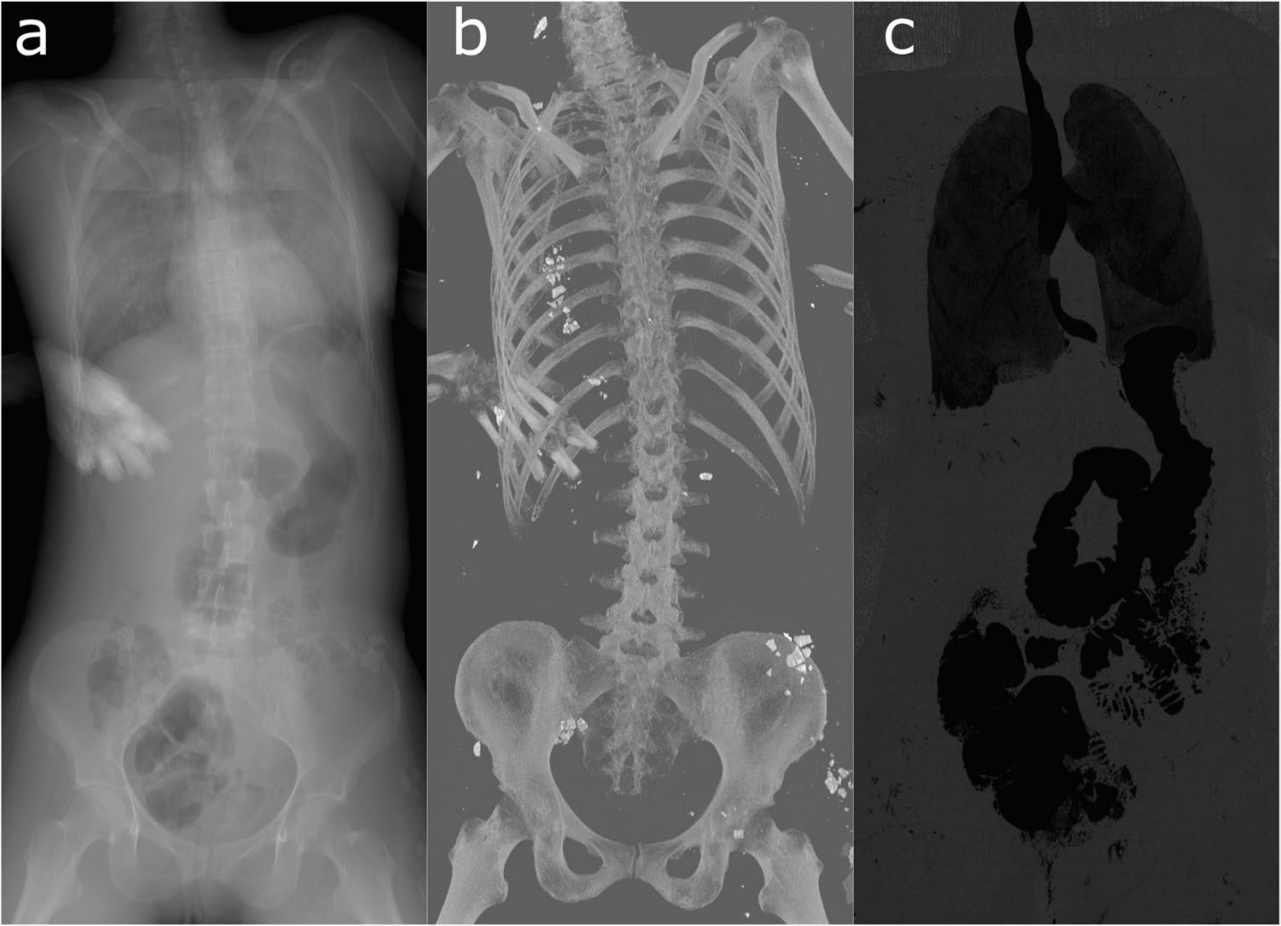
Different volume visualizations of the same PMCT dataset. **a** Averaging visually mimics the appearance of a plain radiograph. **b** MIP highlighting structures with high x-ray density such as bone. The high-density material visible in this image is debris that is located underneath the body. **c** MinIP visualizing the gas distribution inside the body and excluding the air surrounding the body

MIP and MinIP are two additional visualization methods that use the data in a defined volume to generate a 2D image. In MIP, the voxel with the highest attenuation value on every slice throughout the selected volume is projected onto a 2D image [41], which means that MIP emphasizes high-density materials such as bone and metal. This method can be used in the localization and determination of foreign bodies, for example, in identification based on the presence of dental restorative materials [2, 42, 43]. In contrast, in MinIP, the voxel with the lowest attenuation value on every slice throughout the selected volume is projected onto a 2D image [44], thereby emphasizing low-density structures such as gases. This method contributes, for example, to the visualization of gas embolism, emphysema, or decompositional gas [45, 46] (Fig. 5). MinIP can also be used to evaluate other structures with large differences in attenuation, such as in the display of fluid-filled bronchi in cases of drowning [47].

In addition to MIP and MinIP, when projecting the average attenuation of the voxels throughout the selected volume, a conventional radiograph is approximated that can be used in the creation of a virtual orthopantomogram from PMCT data [48].

### Volume visualizations

**Fig. 6:**
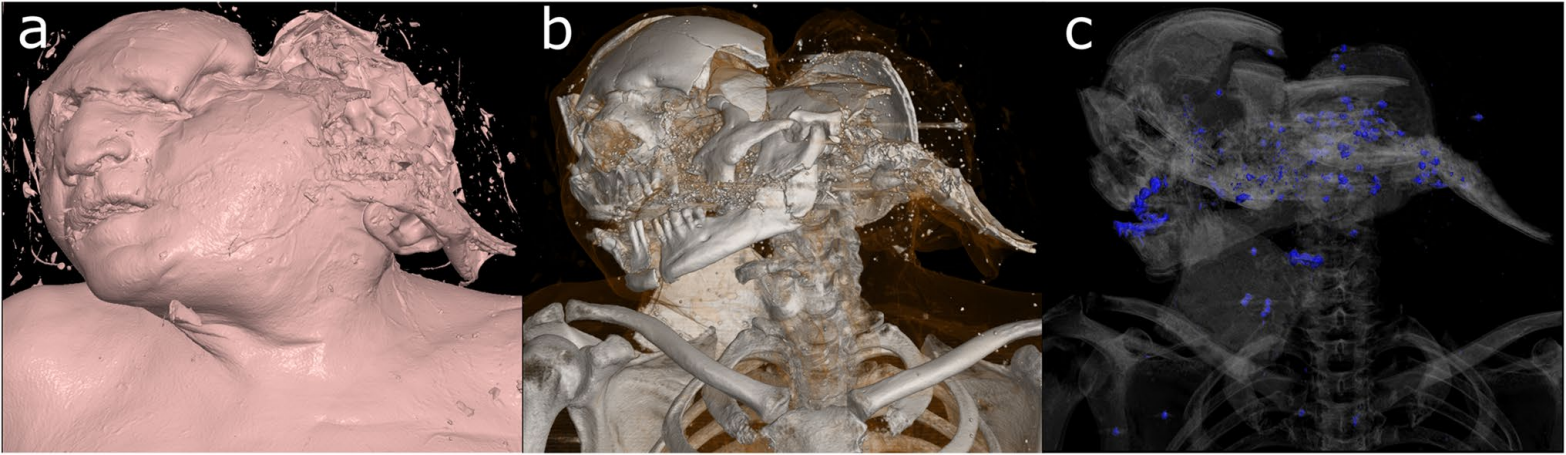
VRT of a suicidal shotgun injury demonstrating visualization using different transfer functions. (**a**) Surface visualization. (**b**) Bone visualization. (**c**) Visualization of radio dense material in blue

Frequently, 2D visualizations may be supplemented by 3D visualizations. An early technique allowing for such a 3D visualization was surface-shaded displays (SSD); this technique only visualizes voxels within a defined Hounsfield range, uses a simplistic lighting model, and allows real-time rendering. SSD have been mostly replaced by volume-rendering techniques (VRT) in most medical image viewers due to the increasing processing power available. Similar to windowing in 2D images, in which HU are mapped to grayscale values, in VRT a voxel is rendered by mapping HU to color and opacity values [41]. This mapping is called a transfer function. By defining appropriate transfer functions, specific structures can be highlighted or diminished in the final presentation (Fig. 6). Furthermore, a variety of tools, such as clipping planes and punch tools, allow the user to remove parts of the volume dataset to achieve a better depiction of the desired finding.

Volume rendering can create a simplified pictorial representation of significant findings in 3D [49]. Yet, while visually pleasing, volume-rendered models alone are often too imprecise for use in the assessment of CT data [23]. When used as a supplement to 2D visualizations, they enable a quick overview (i.e., fracture patterns, foreign bodies) in the initial analysis process, a capability that is specifically useful in high-resolution datasets [23]. Furthermore, they are visually more accessible to medical laypersons (i.e., for presentation in a courtroom) and are considered the preferred visualization method by state attorneys [24, 31].

**Fig. 7:**
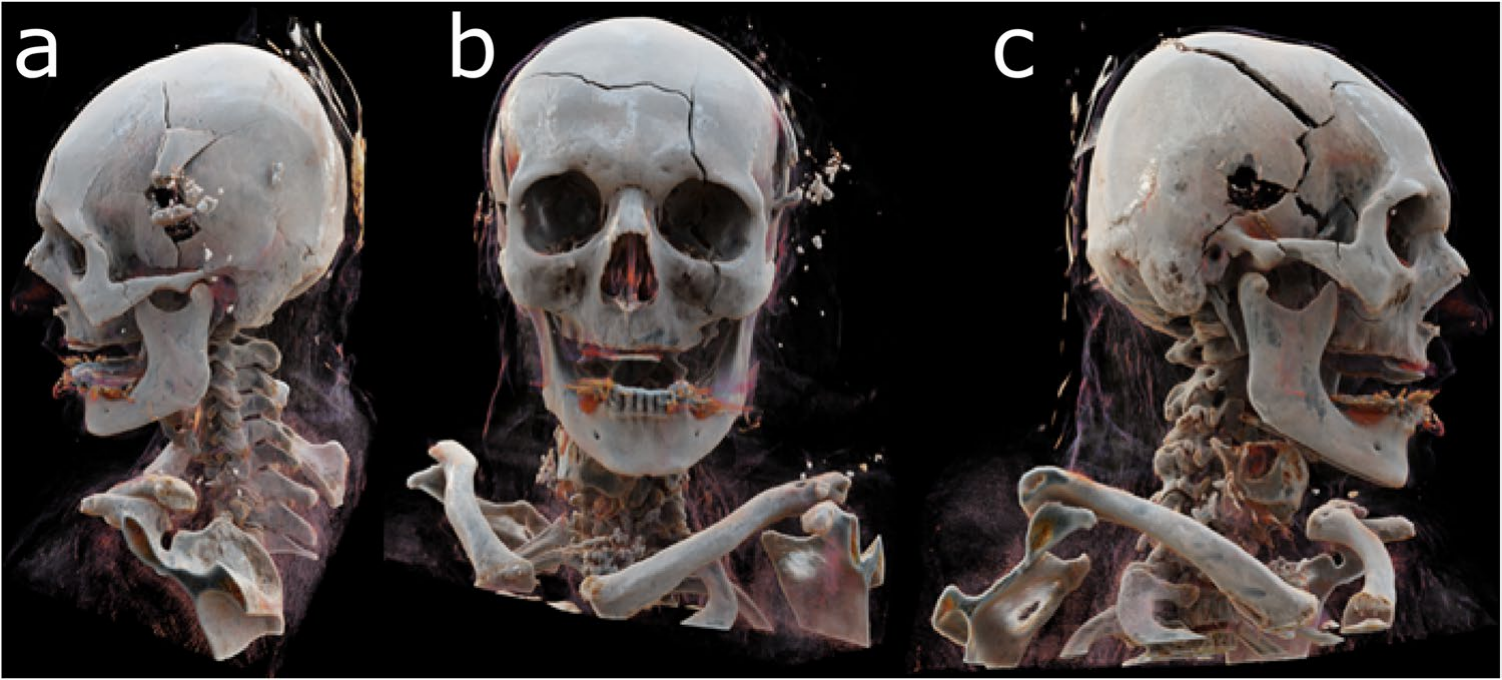
Cinematic rendering of a suicidal gunshot injury to the head. The rendering demonstrates increased perceived realism through the physically correct calculation of optical effects such as shadowing. **a** View from the left showing the exit wound. **b** Frontal view with skull fracture. **c** View from the right with entry wound

More recently, new sophisticated rendering algorithms even permit the simulation of photon behavior, resulting in the correct calculation of optical effects such as depth-of-field and shadowing. These techniques promise more realistic visualization and better depth perception, but this is achieved at the expense of rendering speed [50] (Fig. 7). In general, low-resolution data or data containing image artifacts will result in insufficient visualizations, especially in 3D [34].

Visualization, specifically when 3D rendering is used, also holds certain dangers in forensics. Realistic-looking images and models will be more readily accepted as valid without further questions, as evidenced by Siemens Healthineers release of Cinematic Rendering. This phenomenon of “if it looks plausible, it is plausible” is called naïve realism [51, 52] and should always be kept in mind when creating visualizations to avoid undue influence or prejudice.

## Segmentation

Segmentation is the principal tool used to generate 3D polygon models of specific anatomical structures (such as a whole organ) from 2D CT slices. Using various techniques, each voxel is assigned a label that allows the differentiation of tissues, organs, and anatomical structures. Depending on the method (manual, semiautomatic, or automatic), the accuracy of this tool is to a certain degree influenced by the experience of the operator [53]. Successful use of segmentation requires expert knowledge of anatomy and pathology as well as knowledge of the available tools. Lack of expertise might introduce serious errors in the segmented volume and in the subsequent assessment. In addition, all visualization techniques are highly dependent on the quality and resolution of the data [54]. Another disadvantage of manual segmentation is that it is a rather time-consuming and labor-intensive process. Semiautomatic methods such as region growing [55], which automatically selects all voxels within a defined Hounsfield range that are connected to a seed point specified by the operator, are used to speed up the segmentation process. Still, even fully automatic segmentation methods require some sort of external influence by the operator, such as in the selection of initial parameters, and this significantly affects the model outcome [56].

**Fig. 8:**
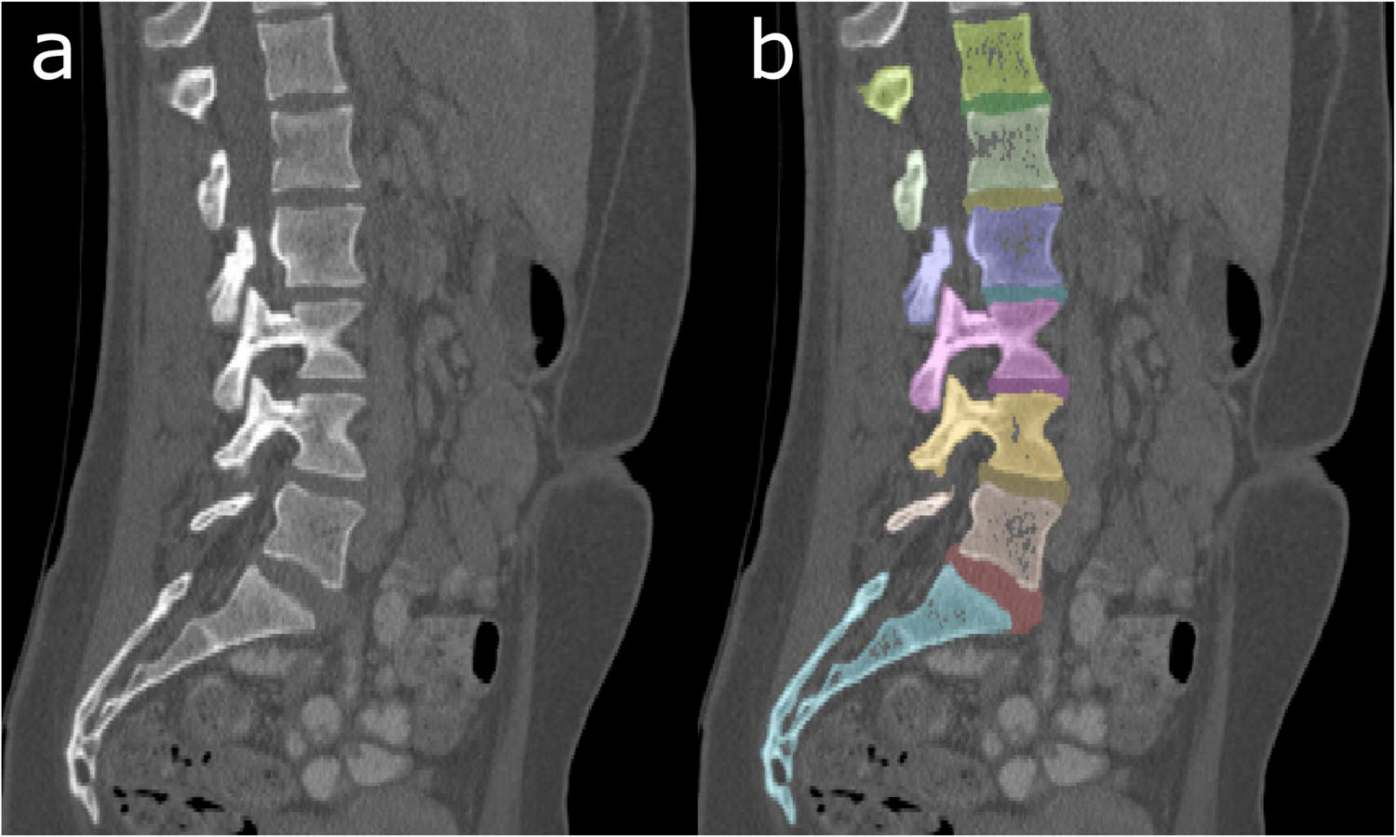
Segmented data. **a** Sagittal view of a PMCT dataset. **b** Segmented spine. Each vertebra, disc, and the sacrum has been semiautomatically segmented and color-coded

Usually, a combination of different segmentation tools and methods is used to perform a segmentation task. The most common methods are thresholding, region growing, livewire, classifiers, clustering, machine learning, and atlas-guided approaches [57–59]. Examples of segmented and unsegmented datasets can be seen in Fig. 8.

In thresholding, a defined range of attenuation values (HU) is identified in all pixels to highlight a desired structure. Thresholding is primarily used in the visualization of findings with a high attenuation difference from the surrounding tissue, such as bones, contrast agents, or metallic foreign bodies. Thresholding is of limited use in the segmentation of parenchymal organs and soft tissue lesions due to their small differences in attenuation values. The operator must define the range of attenuation values individually accordingly to the structures that are to be imaged.

Region growing is often used to further identify a desired structure that is predefined by an initial other segmentation method (mostly thresholding). Region growing checks for interconnectivity using the predefined parameters and then isolates an object of interest. Region growing can also be dynamic where a seed point is selected by the modeler. The segmentation expands from the seed point to the neighboring pixels based on the user’s criteria, such as intensity or a gradient. The segmentation process continues to expand until the neighboring pixels no longer satisfy the initial criteria [56].

Livewire segmentation is one type of semiautomatic method in which the user clicks on the object of interest and draws a delineation around it [60], which can be done along any of the two planes of view and will automatically segment the third plane. This method can speed up the process of segmenting structures by using a contour that is visible to the operator.

Classifiers rely on pattern recognition to isolate structures in a feature space. They require training data from manually segmented sources to use as a reference for automatically segmenting new data. The creation of these training sets relies on manual modeling and therefore is subject to observer error. Clustering is very similar to classifier methods but does not utilize training data. Clustering does, however, require initial input from direct segmentation, but it does not distinguish well between regions of interest and is sensitive to noise and image inhomogeneities [57].

More advanced methods of image segmentation include machine learning approaches, atlas-based approaches, or statistical shape modeling (SSM), all of which aim towards the ultimate goal of fully automatic segmentation of features or structures [61–65].

Regardless of the method used, proper segmentation is vital because it is the basis of all resulting 3D data and models. Any resulting work is only as good as the initial imaging capture and segmentation.

## Polygon visualization

Segmentation masks (labeled voxels) can be converted to polygon meshes using techniques such as the marching cubes algorithm [66]. The resulting 3D surface models generated from CT data are only as good as the resolution quality of the image data. Thicker slices may result in stepped or pixellated-appearing models [34]. The surface models are representations of the surface of the actual physical object and are the base format for methods such as morphometrics [67], virtual physics simulation [68], 3D printing [69], and enhanced visualization or animation [16]

Once a 3D model is generated, it can be exported from the software package as an STL, an OBJ, or in another appropriate file format. In all 3D modeling methods, once a 3D mesh is generated, it is rarely left in its initial capture state, and additional processing steps are required. Any holes in the mesh must be patched or filled for it to run properly in other software. The number of triangles of a mesh must be reduced for ease of computation. Various algorithms can check and repair a 3D mesh to remove potential issues such as intersecting or overlapping triangles or poor triangle quality. Artifacts of the scanning process also need to be removed. Noise reduction will be handled through global, local, or manual smoothing methods. Noise shells, stray bits of mesh that do not contribute to the object, need to be removed. It is possible to convert 3D polygon meshes into 3D PDFs that can be viewed using standard software [70], which offers a means to provide 3D data to prosecutors and judges.

## Mixing and layering

**Fig. 9:**
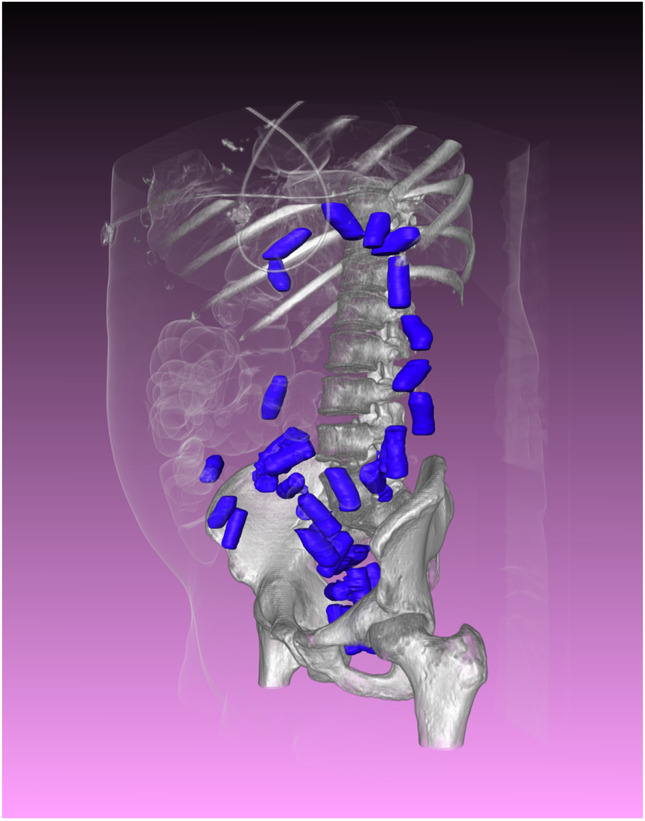
Visualization of drug containers on PMCT in a case of body backing. VRT of soft tissue and bone was mixed with a polygon rendering resulting from segmentation of the drug containers

**Fig. 10:**
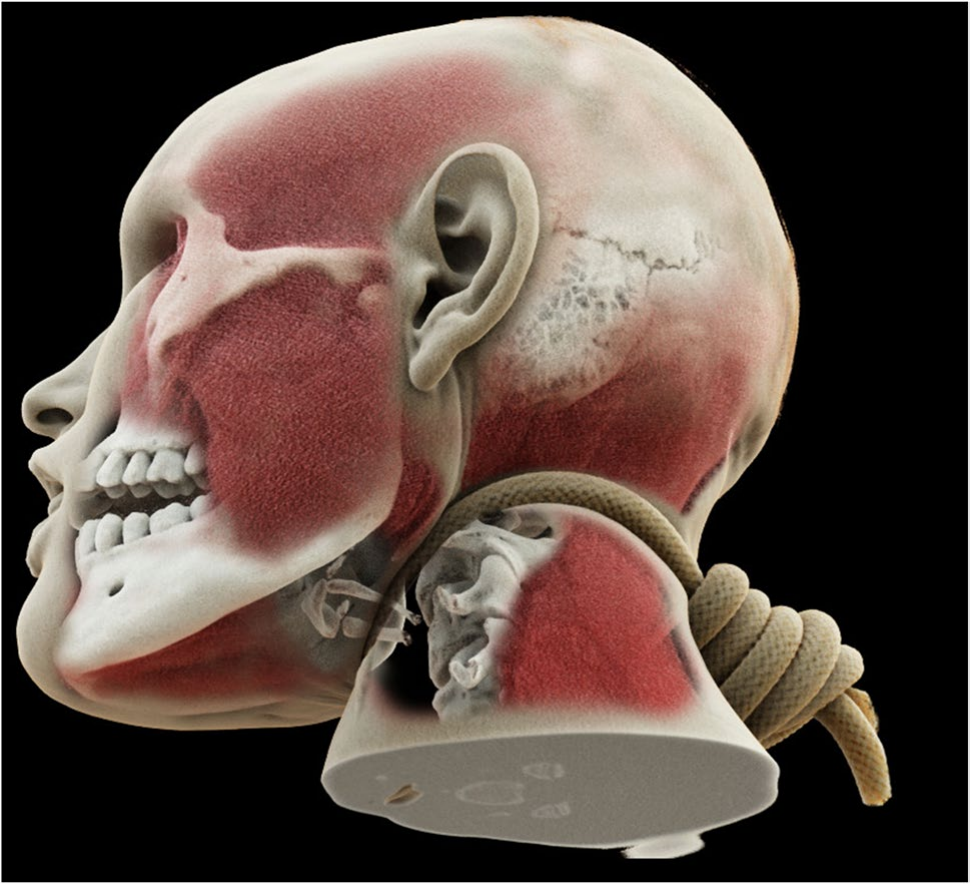
Visualization of a hanging using multiple layered cinematic renderings (bone, muscle body, and surface). Using this method, the bony structures of the neck and the rope can be visualized at the same time despite the fact that the rope has a Hounsfield density similar to that of the soft tissue. Painted transparencies on different layers allow exposure of underlying structures such as the hyoid bone

Each visualization method has its respective advantages and disadvantages. It is, however, possible to employ multiple methods to achieve the desired result. For example, polygon rendering and VRT can be used in conjunction (Fig. 9). It is also possible to incorporate additional information. As an example, small CT-dense particles may be projected onto 3D surfaces and combined with an SSD or VRT reconstruction to allow better visual recognition in combination with anatomical references [71]. An alternative to mixing different visualizations is layering of multiple VRTs that have been rendered using different transfer functions (Fig. 10).

## Annotations and highlighting of image reports

**Fig. 11:**
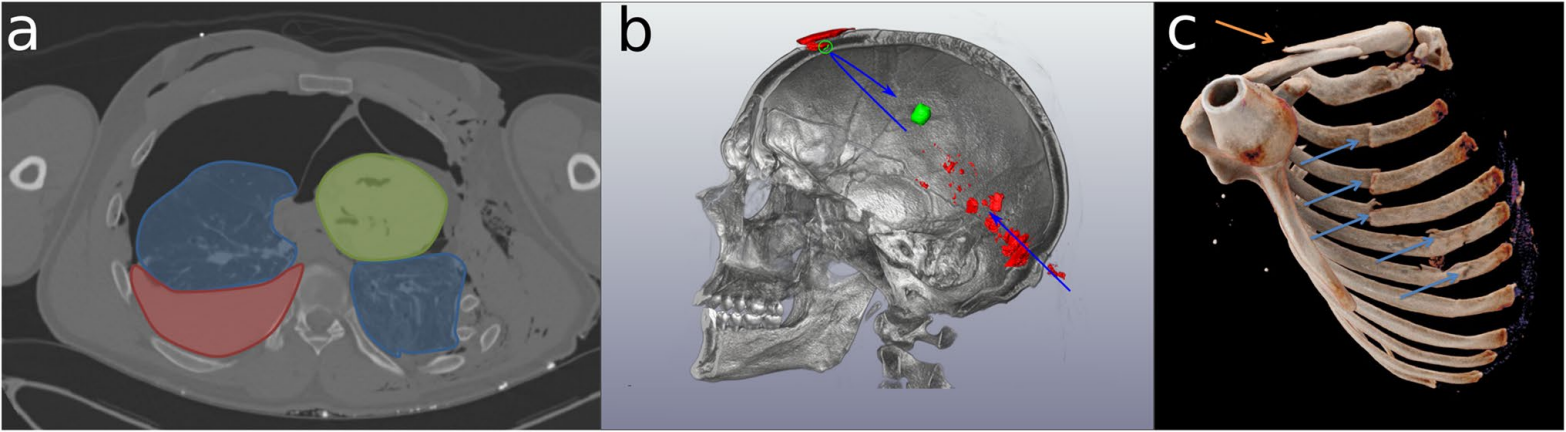
Annotated images. **a** Visualization of hemothorax and tension pneumothorax by color-coding of different structures. Heart (green), lungs (blue), and blood (red). **b** Combined VRT and polygon rendering displaying dislodged bone fragments. Additional annotations highlight the bullet path as well as a smaller projectile fragment. **c** Cinematic rendering of fractures of the ribs and clavicular bone, annotated using different-colored arrows

Especially for medical laypersons, 2D slides extracted from cross-sectional imaging datasets may be difficult to understand. The anatomical context is often not clear, and the lack of actual colors can add to the problem. Proper annotation of 2D images, including color coding, markers, and arrows, is usually not available in medical visualization software but can be added later using software such as LibreOffice Impress or Inkscape (Table 1). In 3D visualizations, annotations can help localize specific findings (Fig. 11).

## 3D printing

**Fig. 12:**
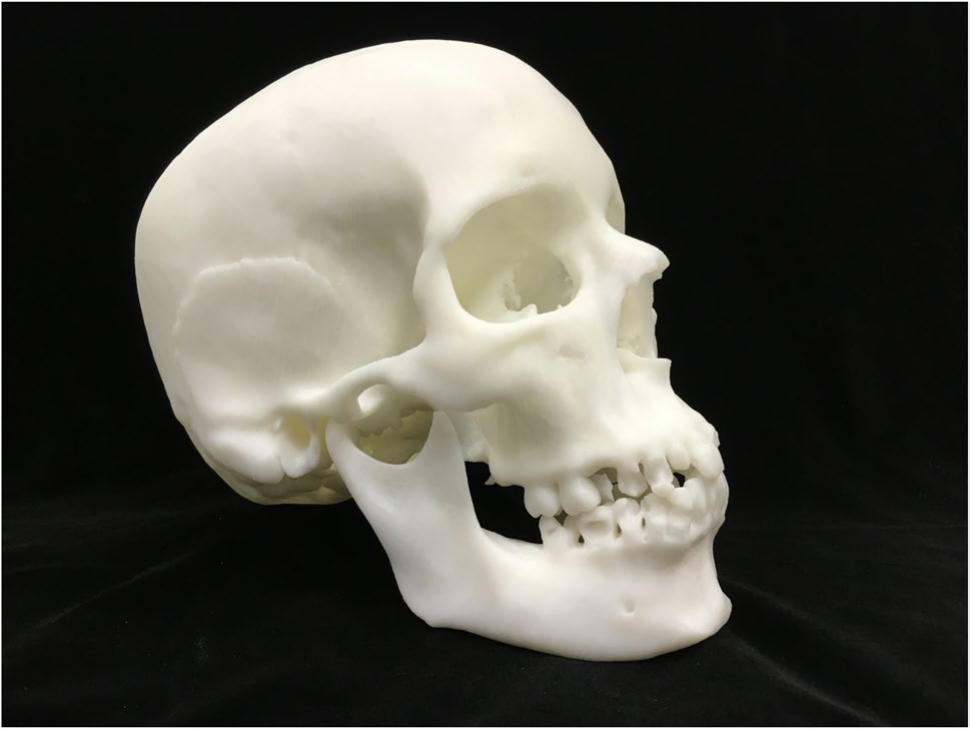
3D-printed skull extracted from a CT scan using FDM printing

3D printing is a rapid prototyping technique that allows the physical creation of an object based on digital information (Fig. 12). A replica of any object that has been captured in 3D may be produced via 3D printing. 3D printing makes it possible to analyze and present objects in a way that originally could only be accomplished via destructive means [72]. The ability to create duplicate 3D structures in another material allows the creation of artificial replicas of funerary and forensic remains. The application of this technology is limited only by its availability, by the user’s ideas, and, of course, by the technical limitations of the software and 3D printing devices used. 3D printing today has gone beyond making device prototypes. Many printers will print to the micron level, making them valuable to forensic practitioners [73] and even in clinical applications for diagnostic and surgical planning purposes.

There are countless professional and hobbyist 3D printers on the market. Each printer uses either some sort of fused deposition modeling (FDM), stereolithography (SLA), selective laser sintering (SLS), selective laser melting (SLM), or electronic beam melting (EBM). FDM uses a variety of materials, the most common being PLA or ABS plastics [27]. To create the print, it heats the thermoplastic material so that it will flow and then builds an object layer by layer. Figure 8 shows an image of a skull that was printed using FDM.

SLA technology uses a liquid resin that interacts with light/lasers to solidify each layer to create the desired object. SLS uses a laser to heat powdered metal so that it fuses with its neighbors to form the desired shape. SLM is similar to SLS, but instead of heating the metal to the point of fusion, SLM melts it so that it combines with its neighbor. EBM uses similar technology, but instead of lasers, it uses an electron beam. The types of materials utilized in these 3D printers include plastics, resins, glass, ceramics, and metals. 3D printing is not cheap, but there are affordable desktop varieties available such as MakerBot, FormLabs, and 3D Systems. The larger and more expensive SLS machines are usually limited to large industrial manufacturers. However, outsourcing of 3D printing to commercial facilities is always an option if in-house printing is not feasible, taking into account data privacy.

The forensic community uses 3D printing technology in various applications [74]. This includes forensic facial reconstruction [75], weapon matching [76], presentation of evidence in court [69, 77], anthropological evidence reconstruction [78, 79], and educational purposes in a classroom setting [80]. As an additional application, 3D printing can be used to quickly manufacture workpart prototypes (rapid prototyping) [81]. Rapid prototyping has relevance in forensics because if allows for the development of custom made parts in low quantities [82].

## Stereoscopic displays/AR/VR

Conventionally, medical imaging data, even when rendered in 3D, are usually displayed as 2D projections and displayed on a screen. This means that scale and depth information is lost on such renderings. Stereoscopic displays that offer a real 3D impression have shown to have advantages in clinical settings such as pre-operative planning or minimally invasive surgery. They can also help in understanding complex 3D structures [83]. When positional and rotational tracking of the users head is added to 3D displays, virtual reality (VR) and augmented reality (AR) applications are possible. While virtual reality creates a scene independent of the physical surroundings, augmented reality provides additional information that is embedded in the real world surrounding the viewer. Visualizing using AR means that the embedding of information can provide a forensic expert with additional information during autopsy, for example, by providing access to image data [84, 85]. In theory, both VR and AR can display volumetric and polygonal data in 3D. However, AR devices usually do not have the processing capabilities required for VRT. Some of the software packages used for medical visualization (such as *3D Slicer*) offer VR support through plugins [86]. Meshes that have been extracted from medical datasets can be incorporated into incident reconstructions that can be visualized in VR for various purposes such as displaying the reconstruction to state attorneys or conducting reconstructions or virtual crime scene visits [87–89].

## Conclusion

PMCT is an image modality that is often used in forensic medicine and forensic incident reconstructions. Case- and audience-specific visualization is vital to identifying relevant findings and communicating them appropriately. A large number of different 2D and 3D techniques, each of which has specific applications, exist. Knowledge of these techniques is essential to successfully handling PMCT data in a forensic setting.

## Data Availability

Not applicable.
